# An extremely high dietary iodide supply forestalls severe hypothyroidism in Na^+^/I^−^ symporter (NIS) knockout mice

**DOI:** 10.1038/s41598-017-04326-z

**Published:** 2017-07-13

**Authors:** Giuseppe Ferrandino, Rachel R. Kaspari, Andrea Reyna-Neyra, Nabil E. Boutagy, Albert J. Sinusas, Nancy Carrasco

**Affiliations:** 1 Department of Cellular and Molecular Physiology, Yale School of Medicine, New Haven, CT 06510 USA; 2 Section of Cardiovascular Medicine, Department of Medicine, Yale School of Medicine, New Haven, CT 06510 USA; 3 Department of Radiology and Biomedical Imaging, Yale School of Medicine, New Haven, CT 06510 USA

## Abstract

The sodium/iodide symporter (NIS) mediates active iodide (I^−^) accumulation in the thyroid, the first step in thyroid hormone (TH) biosynthesis. Mutations in the *SLC5A5* gene encoding NIS that result in a non-functional protein lead to congenital hypothyroidism due to I^−^ transport defect (ITD). ITD is a rare autosomal disorder that, if not treated promptly in infancy, can cause mental retardation, as the TH decrease results in improper development of the nervous system. However, in some patients, hypothyroidism has been ameliorated by unusually large amounts of dietary I^−^. Here we report the first NIS knockout (KO) mouse model, obtained by targeting exons 6 and 7 of the *Slc5a5* gene. In NIS KO mice, in the thyroid, stomach, and salivary gland, NIS is absent, and hence there is no active accumulation of the NIS substrate pertechnetate (^99m^TcO_4_^−^). NIS KO mice showed undetectable serum T_4_ and very low serum T_3_ levels when fed a diet supplying the minimum I^−^ requirement for rodents. These hypothyroid mice displayed oxidative stress in the thyroid, but not in the brown adipose tissue or liver. Feeding the mice a high-I^−^ diet partially rescued TH biosynthesis, demonstrating that, at high I^−^ concentrations, I^−^ enters the thyroid through routes other than NIS.

## Introduction

Iodine is an essential constituent of the thyroid hormones (THs), which are phenolic rings joined by an ether link and iodinated at either three positions (3,5,3′-tri-iodo-l-thyronine, or T_3_) or four positions (3,5,3′,5′-tetra-iodo-l-thyronine, or T_4_). The THs are required for the proper development of the central nervous system^1^, skeleton^2^, and lungs^3^ in the fetus and the newborn and for intermediary metabolism at all ages^4^. Therefore, both hyper and hypothyroidism are diseased states with serious systemic ramifications. Iodide (I^−^) is an extremely scarce nutrient in the environment and is supplied only in the diet. The Na^+^/I^−^ symporter (NIS), a member of solute carrier family 5 (SLC5), is the plasma membrane protein that actively transports I^−^ into the thyroid follicular cells, using as its driving force the Na^+^ gradient generated by the Na^+^/K^+^ ATPase. NIS transports I^−^ electrogenically with a 2 Na^+^ : 1 I^−^ stoichiometry^1^. Since our group cloned the cDNA coding for NIS^5^, we have reported *in vivo* and *in vitro* that NIS is regulated transcriptionally and post-transcriptionally by thyroid stimulating hormone (TSH)^6–9^. We have identified residues that coordinate NIS substrates^10–12^, and elucidated the basis for the efficient transport of I^−^ by NIS at the physiological (submicromolar) concentrations of I^−^ in the plasma^13^. Besides the thyroid gland, NIS is functionally expressed in other tissues including lactating breast^14^, breast cancer^9, 14^, salivary glands^14^, and stomach^1, 9, 14^.

NIS-mediated I^−^ transport is the key first step in TH biosynthesis, which occurs partly intracellularly and partly in the colloid, an extracellular compartment. I^−^ reaches the colloid through an I^−^ efflux process that has not been fully elucidated. The genetic characterization of patients affected by Pendred syndrome, a disorder characterized by goiter and a partial I^−^ organification defect^15^, led to the identification of pendrin (*SLC26A4*) as a protein that may mediate I^−^ efflux into the colloid. However, pendrin knockout (KO) mice do not display hypothyroidism^16^, indicating that other mechanisms are also involved in thyroid apical I^−^ efflux. The Na^+^/monocarboxylate transporter (SMCT) (*Slc5a8*) is expressed at the apical membrane of thyroid follicular cells^17^. SMCT was initially proposed to mediate I^−^ efflux into the colloid, because of its high sequence similarity to NIS (~70%)^18^. However, we demonstrated that SMCT does not transport I^−^^17^ and *Slc5a8* KO mice are not hypothyroid^19^. The calcium-activated chloride channel anoctamin1 (*Ano1*) is expressed at the apical membrane of thyroid follicular cells^20^. HEK 293 T and PCCl3 cells co-transfected with plasmids encoding NIS and *Ano1* show increased I^−^ efflux in the presence of ionomycin, a Ca^+^ ionophore, suggesting that *Ano1* may play a role in I^−^ efflux^20^. *Ano1* KO mice die within their first month of life and displayed severe tracheomalacia, with gaps in the cartilage rings all along the trachea^21^. Independently of how I^−^ crosses the apical surface, once it reaches the colloid, it is oxidized to iodine and covalently incorporated into thyroglobulin (TG) by dual oxidases (DUOX) 1 and 2 and thyroperoxidase (TPO)^1^. TH biosynthesis and release is stimulated by TSH, which is produced by the pituitary gland. TSH binds to its receptor, which is located at the basolateral membrane of the thyroid follicular cells, and stimulates the endocytosis and proteolytic cleavage of iodinated TG, resulting in the release of T_3_ and T_4_ into the bloodstream^22^. Iodine in uncoupled monotyrosine (MIT) and diiodotyrosine (DIT) is reutilized by DEHAL, an iodotyrosine deiodinase that catalyzes the dehalogenation of mono- and diiodotyrosine, allowing I^−^ to be recycled for further TH biosynthesis.

The total or partial impairment of NIS function due to mutations in the *SLC5A5* gene (which encodes NIS) has long been known to cause congenital I^−^ transport defect (ITD), which, if left untreated, leads to stunted growth and cognitive deficits^1^. ITD follows an autosomal recessive inheritance pattern and is diagnosed by reduced or absent thyroid I^−^ uptake and a low saliva-to-plasma I^−^ ratio (normal value is >30). ITD is clinically characterized by hypothyroidism, goiter, and mental impairment of varying degrees. However, there are reports of a few ITD patients whose I^−^ intake was unusually high (~100 times higher than the 150 µg/day recommended by the World Health Organization) and whose hypothyroidism was less severe^23, 24^. This suggests that, in the absence of a functional NIS, high dietary I^−^ supply may result in some entry of I^−^ into the follicular cells through non-NIS paths, leading to at least modest TH biosynthesis.

We have generated a NIS KO mouse model and used it to test the hypothesis that high concentrations of I^−^ in the plasma, resulting from high dietary I^−^, make it possible for I^−^ to enter the thyroid follicular cells in the absence of NIS. Existing animal models of hypothyroidism rely on the use of propylthiouracil (PTU) or methimazole (MMI)^25–27^; these are well-known antithyroid agents that inhibit I^−^ organification, but also have side effects^28–30^. Thus, we took advantage of our NIS KO model to analyze, under conditions free of pharmacological manipulation, some endocrine and metabolic alterations resulting from the hypothyroid state of these mice.

## Results

### NIS KO mice on a standard chow diet (CD) produce low levels of T_4_ but have unaltered levels of T_3_

**Figure 1:**
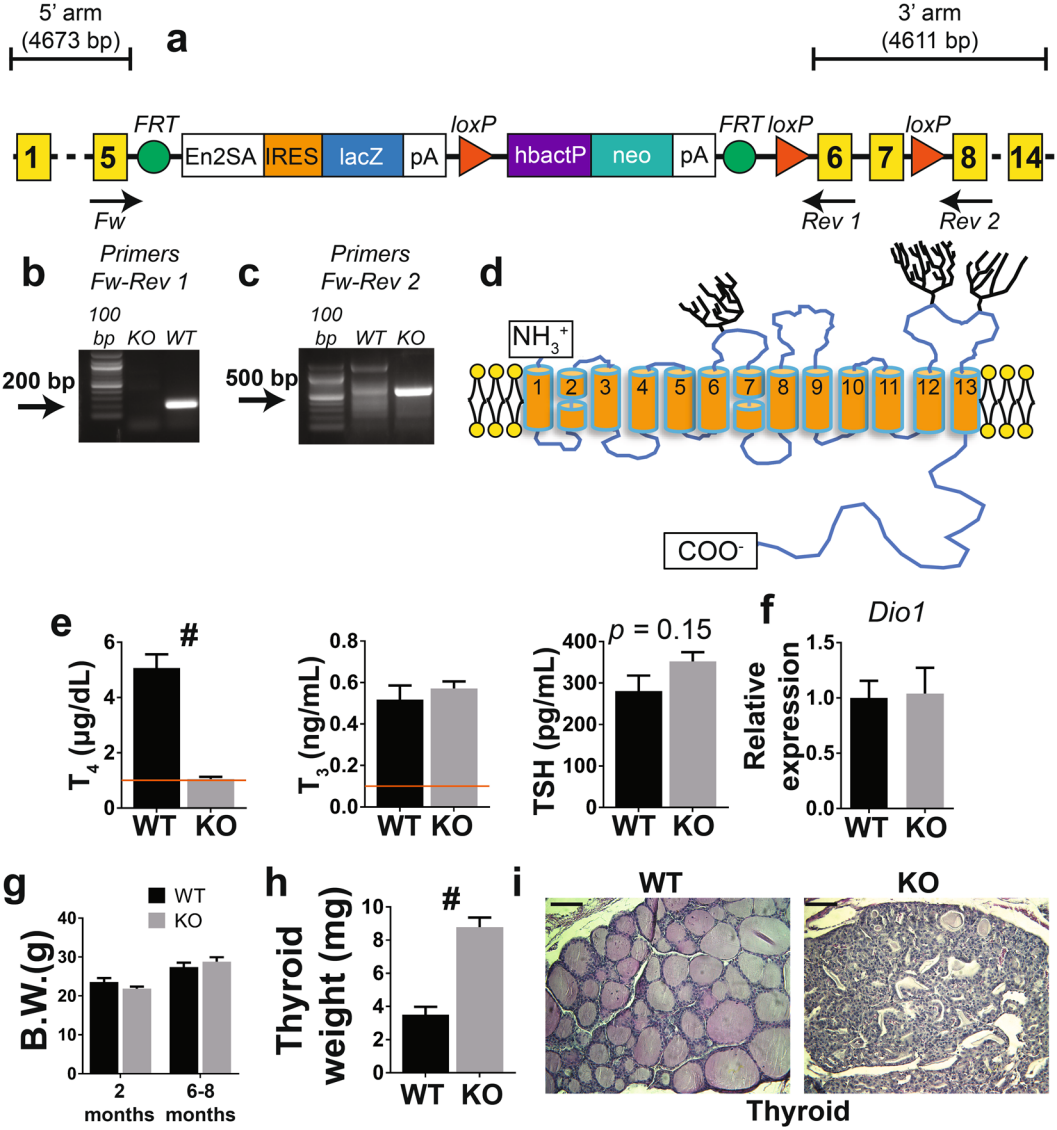
Schematic representation of the targeted region of the *Slc5a5* gene, which codes for NIS, and metabolic characterization of WT and NIS KO mice fed a CD. (**a**) The targeted *Slc5a5* allele. Yellow squares mark the exons. (**b**) The PCR product obtained using the primers Fw and Rev 1. NIS KO mice, which lack exons 6 and 7, do not show any PCR product, while WT mice show the expected 200 bp amplicon. (**c**) The PCR product obtained using the primers Fw and Rev 2. After *FRT* and Cre recombination, a 700-bp PCR product is obtained in NIS KO mice, due to the excision of exons 6 and 7. (**d**) NIS secondary structure model showing the 13 transmembrane segments (TMSs) and carbohydrates (trees). The targeted exons (6 and 7) code for TMSs 7 and 8. (**e**) Serum TH and TSH levels in 6–8-month-old male WT and NIS KO mice fed a CD. The horizontal line indicates the detection limit of the T_4_ and T_3_ ELISA kits (n = 4–5). (**f**) Liver *Dio1* mRNA expression levels (n = 5). (**g**) Body weight of male WT and NIS KO mice 2 and 6–8 months old fed a CD (n = 5). (**h**) Thyroid weight of 6–8-month-old male WT and NIS KO mice fed a CD (n = 5). (**i**) Hematoxylin and eosin staining of thyroid sections obtained from the dissected thyroids in h. Scale bar = 50 µm. En2SA = engrailed 2 gene splice acceptor, IRES = internal ribosome entry site, pA = polyadenylation, hbactP = human beta actin promoter, neo = neomycin resistance. ^#^Indicates *p* < 0.01.

To generate a constitutive NIS KO mouse model, we obtained embryonic stem cells from the knockout mouse project (KOMP) repository. Gene targeting was used to modify the *Slc5a5* gene (Fig. 1a), which encodes NIS. By homologous recombination, a cassette containing a polyA signal was inserted after exon 5, while exons 6 and 7 were flanked by loxP sites (Fig. 1a). The resulting allele was *Slc5a5* ^*TM1a*^ in KOMP nomenclature. A fully verified clone was injected into blastocysts to generate chimeric mice, which transmitted the *Slc5a5* ^*TM1a*^ allele to their offspring. These chimeric mice were crossed with mice expressing flippase ubiquitously to remove the LacZ, neo, and polyA signal cassettes. An additional crossing with mice expressing Cre ubiquitously was necessary to remove NIS exons 6 and 7 (Fig. 1a). The expected PCR products were amplified from genomic DNA extracted from wild-type (WT) and NIS KO mice (Fig. 1b,c). Exons 6 and 7 encode transmembrane segments (TMSs) 7 and 8 (Fig. 1d), which are crucial for NIS function^12^. Moreover, if exon 8 were to splice with exon 5, Cre recombination would generate a frame shift in exon 8, which would produce a stop codon and hence a truncated NIS protein. Mice heterozygous for the disrupted NIS allele were crossed, and the resulting litters displayed Mendelian ratios.

We then determined serum T_4_, T_3_, and TSH levels in NIS KO mice fed a standard CD and compared the results to data from age- and sex-matched WT mice with the same genetic background (Fig. 1e). Surprisingly, NIS KO mice produced T_4_ and T_3_. T_4_ levels were lower (1.05 ± 0.07 µg/dL) than in WT mice (5.05 ± 0.6 µg/dL), while T_3_ levels (0.51 ± 0.06 ng/mL) were similar to those in WT mice (0.56 ± 0.02 ng/ml). TSH values were higher (albeit non-significantly) in NIS KO mice (352 ± 22 pg/mL) than in WT animals (280 ± 37 pg/mL) (Fig. 1e). The unaltered T_3_ levels in the serum of NIS KO mice fed a CD may be explained by the observation that D1^31^ was expressed at the same levels in the livers of NIS KO mice and WT mice (Fig. 1f). There were no differences in body weight between NIS KO and WT mice either at 2 or at 6–8 months of age (Fig. 1g). The thyroids of NIS KO mice were larger (8.8 ± 0.6 mg) than those of WT mice (3.5 ± 0.5 mg) (Fig. 1h), a finding consistent with the low T_4_ and high TSH levels in NIS KO mice. The characteristic follicular structure was lost in the thyroids of 6–8-month-old NIS KO mice, as shown by hematoxylin and eosin-stained sections of these glands (Fig. 1i).

That the NIS KO mice did produce some T_4_ could be explained in one of two ways: 1) NIS KO mice were not successfully generated, so that some residual NIS was present to mediate I^−^ uptake, or 2) if NIS KO mice were successfully generated, I^−^ entered the thyroid follicular cells by routes other than NIS.

### TH biosynthesis still occurs in NIS KO mice, but not owing to residual NIS activity

**Figure 2:**
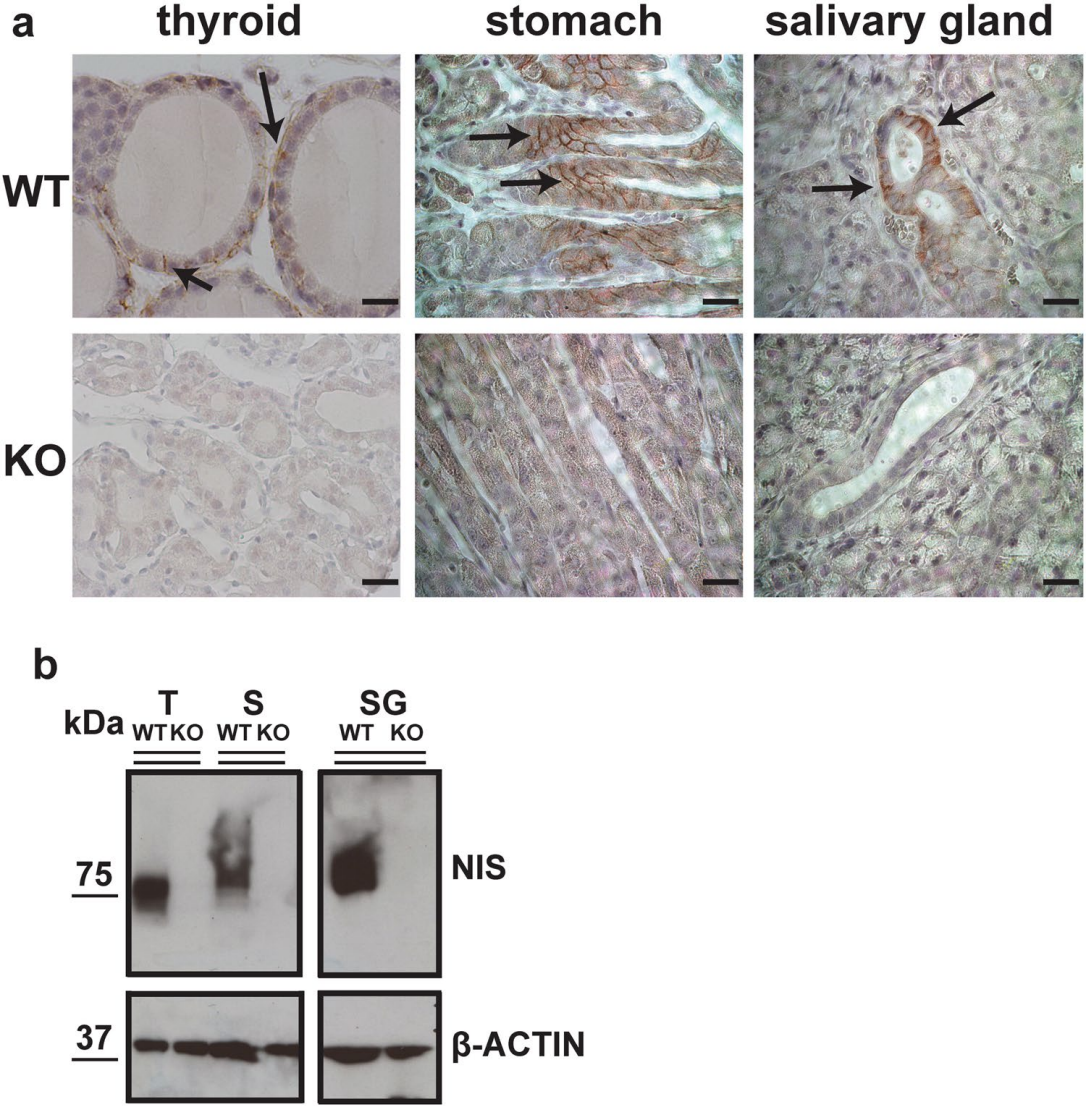
NIS protein expression is abolished in NIS KO mice. (**a**) Sections of thyroid, stomach, and salivary glands obtained from WT or NIS KO mice fed a CD were incubated with anti-NIS Ab and IHC performed as described in Materials and Methods. Arrows indicate the typical basolateral NIS staining observed in organs from WT mice. NIS KO mice did not show any specific staining. Scale bar = 10 µm. (**b**) WB analysis of NIS expression in organs from WT and NIS KO mice. Images were cropped from the full images presented in Supplementary Figure 1.

To determine whether NIS was indeed deleted in our NIS KO mouse model, we investigated NIS expression in NIS KO and WT mice by immunohistochemistry (IHC) and western blot (WB) in the thyroid, salivary glands, and stomach. As expected, in WT mice, IHC showed typical NIS staining at the basolateral plasma membrane of the thyroid follicular cells, mucin-secreting cells in the stomach, and salivary ductal cells (Fig. 2a). In stark contrast, NIS KO mice did not display any specific staining in these three tissues, suggesting that the protein was not expressed there (Fig. 2a). These findings were confirmed by the WB results: there was no immunoreactivity in tissues from NIS KO mice, whereas the typical polypeptide bands corresponding to NIS were evident in the tissues from WT mice (Fig. 2b and Supplementary Figure 1). The electrophoretic mobility of NIS is different in the thyroid, stomach, and salivary glands because, as we have reported previously^32^, NIS is glycosylated to a different extent in each of these three tissues (Fig. 2b).

**Figure 3:**
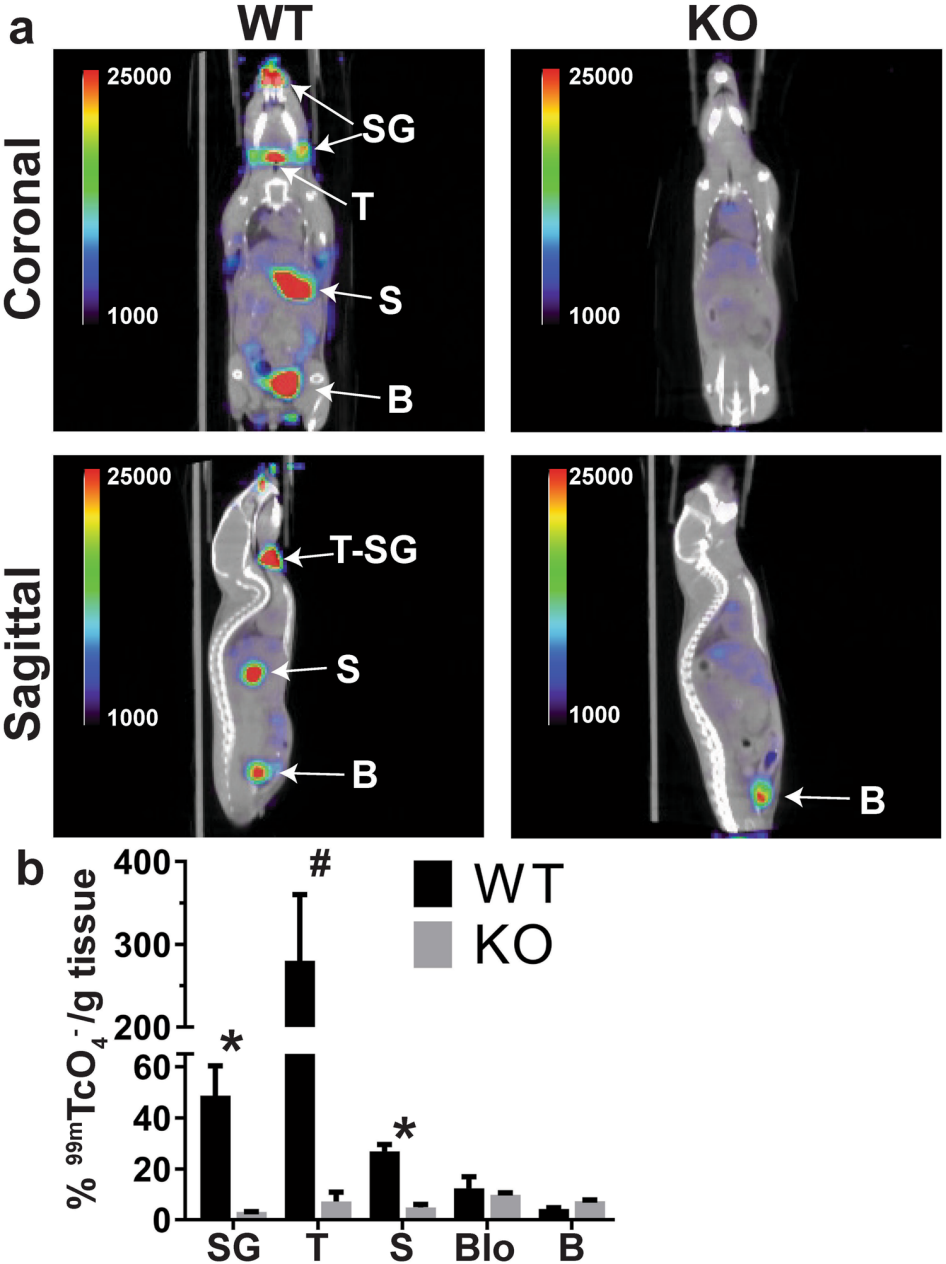
Lack of active accumulation of a NIS substrate in NIS KO mice. (**a**) SPECT/CT analysis of 3-month-old female WT and NIS KO mice fed a CD (coronal and sagittal views). WT mice accumulate ^99m^TcO_4_^−^ in the thyroid (T), salivary glands (SG), and stomach (S). NIS KO mice accumulate ^99m^TcO_4_^−^ only in the bladder (B). Images are displayed as % injected dose/cc. (**b**) Quantitation of ^99m^TcO_4_^−^ in the corresponding dissected organs 3 hours after injection of the isotope (n = 2–3) with gamma well counting. Activity is expressed as % injected dose/gram of tissue. T = thyroid, SG = salivary gland, S = stomach, Blo = blood, B = bladder. *Indicates *p* < 0.05, ^#^ *p* < 0.01.

To investigate the unlikely possibility that the NIS KO mice produced a truncated functional protein undetectable by WB or IHC, because our NIS antibody recognizes the last 16 residues of the carboxy terminus of NIS^6^ (Fig. 1d), we carried out hybrid SPECT (single-photon emission computed tomography)/CT imaging of WT and NIS KO mice fed a CD using the NIS substrate pertechnetate (^99m^TcO_4_^−^)^33^. ^99m^TcO_4_^−^, a NIS substrate that is widely used in clinical medicine, has several advantages over I^−^ isotopes: it is inexpensive, has a short half-life (~6 h), and is not covalently incorporated into TG in the thyroid. As expected, WT mice accumulated ^99m^TcO_4_^−^ in the regions corresponding to the thyroid and salivary glands, and in the stomach (Fig. 3a,b). In NIS KO mice, ^99m^TcO_4_^−^ was detected only in the bladder, as it is eliminated in the urine (Fig. 3a,b).

In conclusion, NIS KO mice do not accumulate ^99m^TcO_4_^−^ in the tissues where WT mice display NIS-mediated active transport of ^99m^TcO_4_^−^ because NIS KO mice do not express NIS.

### NIS KO mice on a minimal iodide diet (MID) have very low T_4_, low T_3_, and high TSH

The minimum required supply of I^−^ for rodents is 0.15 µg per gram of food^34^. The standard CD provides 6 µg of I^−^ per gram of food (i.e., 40 times the minimum required supply). We hypothesized that such a great excess of I^−^ in the diet would sufficiently increase the plasma I^−^ concentration to make it possible for I^−^ to enter the follicular cells via non-specific mechanisms.

**Figure 4:**
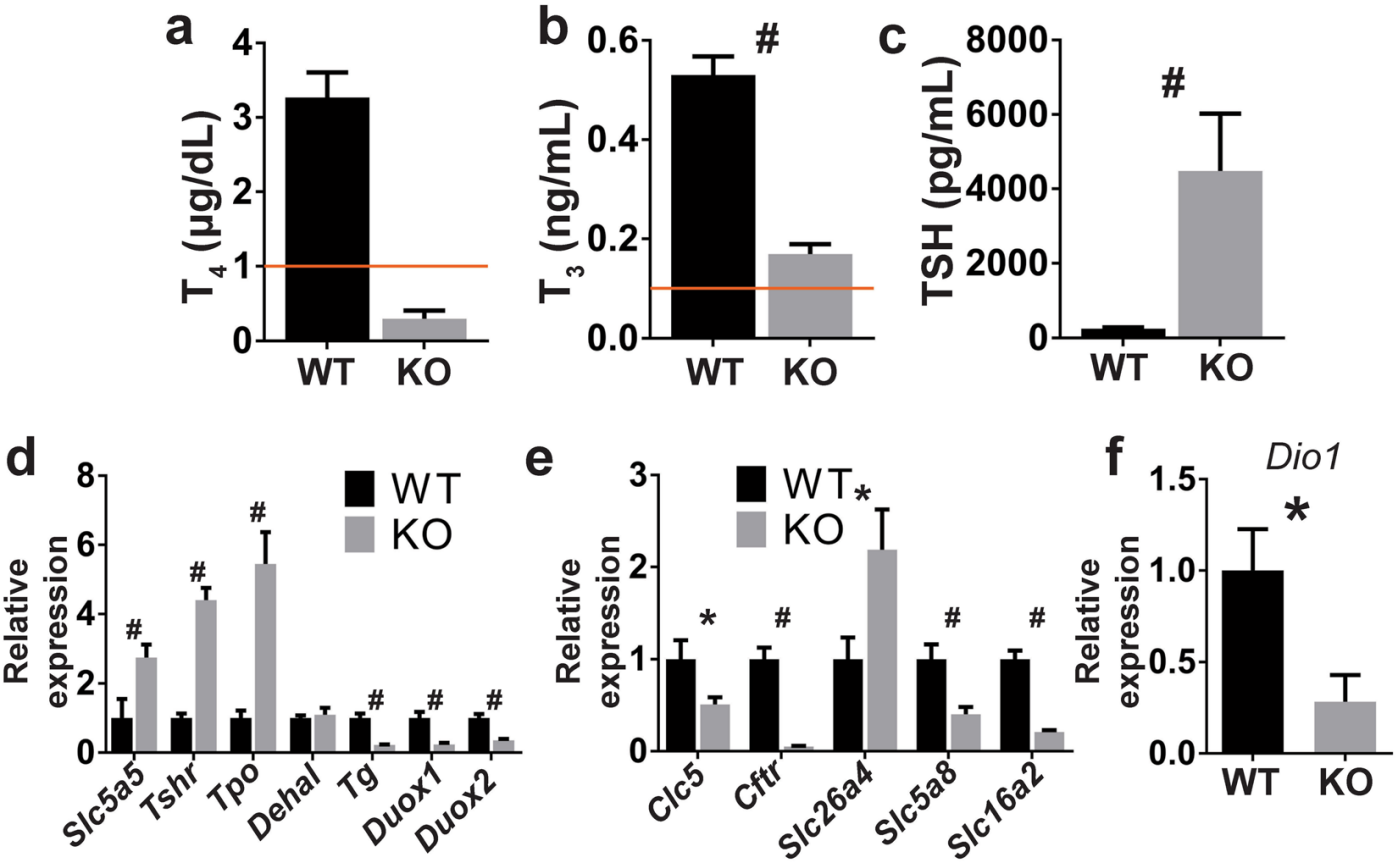
Reduced I^−^ intake impairs TH biosynthesis in NIS KO mice. (**a**,**b**) Serum T_4_ and T_3_ levels of male WT and NIS KO mice fed a MID. The horizontal line indicates the detection limit of the T_4_ and T_3_ ELISA kits. (**c**) Serum TSH levels of male WT and NIS KO mice fed a MID (n = 5). (**d**) Thyroid mRNA expression levels of genes involved in TH biosynthesis (n = 7–8). (**e**) Thyroid mRNA expression levels of apical thyroid proteins. Each group contained 4–5 males and 3 females fed a MID. No sex differences were observed. (**f**) Liver *Dio1* mRNA expression levels in males (n = 7–8). *Slc5a5* encodes NIS, *Tshr* = thyroid stimulating hormone receptor, *Tpo* = thyroid peroxidase, *Dehal* = iodotyrosine deiodinase, *Tg* = thyroglobulin, *Duox 1* and *2* = Dual oxidase 1 and 2, *Clc5* = chloride voltage-gated channel 5, *Cftr* = cystic fibrosis transmembrane conductance regulator, *Slc26a4* encodes pendrin, *Slc5a8* encodes SMCT, *Slc16a2* = solute carrier family 16 member 2, and *Dio1* *=* iodothyronine deiodinase 1. *Indicates *p* < 0.05, ^#^ *p* < 0.01.

To test this hypothesis, we measured T_4_, T_3_, and TSH levels in NIS KO mice fed a MID, which supplies 0.15 µg of I^−^ per gram of food (the minimum recommended amount)^34^. Indeed, these animals had extremely low serum T_4_ levels and low serum T_3_ levels (0.16 ± 0.02 ng/mL) (Fig. 4a,b). In accordance with the reduced serum T_3_ levels, NIS KO mice showed a strong downregulation (~75%) of D1 expression in their liver, compared to WT mice fed the same MID (Fig. 4f). TSH levels were increased in NIS KO mice compared to WT mice fed a MID (Fig. 4c). In conclusion, some THs are biosynthesized in mice totally devoid of NIS as long as their I^−^ supply is sufficiently great to allow I^−^ to enter the thyroid, most likely via non-specific routes.

### TSH-stimulated expression of genes involved in TH biosynthesis is upregulated in NIS KO mice on a MID

I^−^ organification takes place immediately after the anion exits the thyroid follicular cells apically to enter the colloid^22^. I^−^ organification is more efficient under hypothyroid than euthyroid conditions, because high levels of TSH stimulate the overexpression of certain genes involved in TH biosynthesis^22^. NIS KO mice on a MID showed increased expression of the *Tshr*, *Tpo*, and the truncated *Slc5a5* mRNA (Fig. 4d). However, *Tg* and *Duox 1* and *2*, which are not upregulated by TSH^35, 36^, were all strongly downregulated in these mice, as were the apical anion channels *Clc5* and *Cftr*, the apically expressed Na^+^-dependent monocarboxylate transporter (SMCT, encoded by *Slc5a8*), and the basolaterally expressed MCT8 [encoded by solute carrier family 16 member 2 (*Slc16a2*)], whereas the expression of iodotyrosine deiodinase (*Dehal*) did not change in NIS KO mice. Interestingly, *Slc26a4*, which encodes pendrin, was upregulated in NIS KO mice (Fig. 4e), which is consistent with the proposal that pendrin is one of the conduits that facilitate I^−^ transport into the colloid.

### NIS KO mice on a MID exhibit distinct metabolic adaptations in the thyroid, brown adipose tissue (BAT), and liver

**Figure 5:**
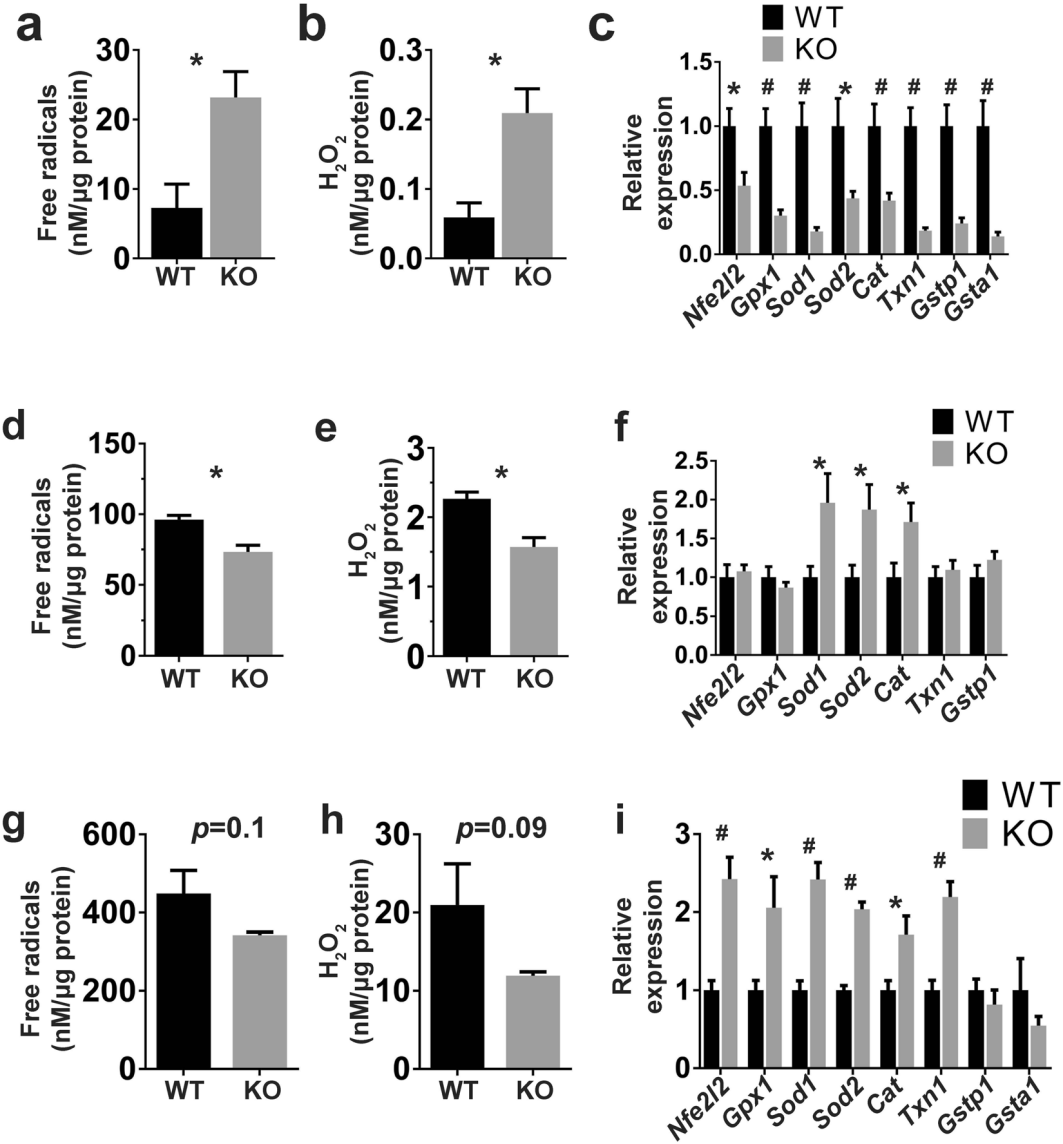
Hypothyroidism produces oxidative stress in the thyroid and a less oxidative intracellular environment in BAT and liver. (**a**,**b**) Quantitation of FR and H_2_O_2_ levels in thyroid extracts from WT and NIS KO mice. (**c**) Expression of genes involved in FR metabolism in the thyroid (n = 7–8). (**d**,**e**) Quantitation of FR and H_2_O_2_ levels in BAT extracts. (**f**) Expression of genes involved in FR metabolism in BAT. (**g**,**h**) Quantitation of FR and H_2_O_2_ levels in liver extracts. (**i**) Expression of genes involved in free radical metabolism in the liver. Each group contained 4–5 males and 2–3 females fed a MID. *Nfe2l2* = nuclear factor, erythroid 2 like 2, *Gpx1* = glutathione peroxidase 1, *Sod 1* and *2* = superoxide dismutase 1 and 2, *Cat* = Catalase, *Txn1* = thioredoxin 1, *Gstp1* = glutathione S-transferase pi 1, and *Gsta1* = glutathione S-transferase alpha 1. *Indicates *p* < 0.05, ^#^ *p* < 0.01.

Reactive oxygen species (ROS) are tightly regulated at multiple levels for homeostasis. In addition, the generation of ROS is affected in a complex fashion by pharmacological intervention. For these reasons, it is often difficult to draw conclusions from studies of ROS. For example, THs have generally been claimed to increase metabolic rates and ROS production^37, 38^; therefore, lower ROS levels are expected in hypothyroidism. However, the antithyroid drug MMI, used to generate hypothyroid mouse models, actually increases oxidative stress^39^. Therefore, our NIS KO mice on a MID represent a unique drug-free model for studying the effect of hypothyroidism on ROS production. NIS KO mice on a MID showed reduced expression of *Duox* genes in the thyroid (Fig. 4d) but, in agreement with previous findings, increased concentrations of H_2_O_2_ and free radicals (FRs) [ROS and reactive nitrogen species (RNS)]^40, 41^ (Fig. 5a,b). Interestingly, the superoxide dismutases (SODs) *Sod1* and *2* (which produce H_2_O_2_); catalase (*Cat*, which disproportionates H_2_O_2_ to H_2_O and O_2_); and *Nfe2l2*, *Gpx1*, *Txn1*, *Gstp1*, and *Gsta1* (genes involved in ROS disposal) were all strongly downregulated in the thyroids of these mice (Fig. 5c). Taken together, these results suggest that TSH stimulation increases oxidative stress in the thyroid by downregulating ROS disposal, because the balance between production and elimination is skewed in the direction of higher ROS levels in the NIS KO mice.

THs stimulate mitochondrial activity^4^. The increased mitochondrial activity leads to higher ROS production in target tissues such as BAT and liver^4, 38, 42^. To study the effect of hypothyroidism on ROS generation in BAT and liver, we measured H_2_O_2_ and FR levels in these tissues. BAT showed a significant reduction in both H_2_O_2_ and FR levels (Fig. 5d,e) as well as upregulation of *Sod1*, *Sod2*, and *Cat* (Fig. 5f). Upregulation of these enzymes may favor the breakdown of H_2_O_2_ into H_2_O and O_2_, thereby reducing H_2_O_2_ levels. Liver tissue, on the other hand, showed a non-significant reduction in H_2_O_2_ and FR levels (Fig. 5g,h), but consistent upregulation of several genes involved in H_2_O_2_ and FR metabolism (Fig. 5i). These results suggest that hypothyroidism causes lower FR levels not only through reduced mitochondrial activity but also via upregulation of genes involved in H_2_O_2_ and FR metabolism.

## Discussion

Mutations in the *SLC5A5* gene, which encodes NIS, lead to congenital hypothyroidism (CH) due to an I^−^ transport defect (ITD). ITD is a rare autosomal recessive condition diagnosed by reduced or absent thyroidal I^−^ uptake and a low saliva-to-plasma I^−^ ratio (<30). To date, 15 ITD-causing NIS mutations have been reported^1^. Invaluable mechanistic and structural information has been obtained by characterizing the amino acid positions bearing mutations in ITD patients and elucidating the molecular requirements of NIS at those positions. For example, the first ITD-causing mutation T354P led us to identify the Na2 binding site of NIS^10, 11, 43^.

Interestingly, the same NIS mutation was discovered in the three children of a Japanese couple, as was the mutation V59E^44^. The patients inherited these two mutations (V59E and T354P) from their healthy mother and father, respectively. The T354P and V59E NIS mutant proteins, when expressed in COS7 cells, were both trafficked to the cell surface, but totally inactive. Surprisingly, the three siblings displayed different degrees of mental retardation. The oldest was nursed for longer than the second oldest, and evinced a less severe cognitive deficit. The youngest was not nursed, and displayed a more severe cognitive deficit than either of her siblings. It was discovered that the mother was addicted to laminaria, an alga extremely rich in I^−^. As NIS is expressed in the lactating breast^14^, where it mediates the transport of I^−^ to the milk, our hypothesis was that breastfeeding the oldest and second-oldest children supplied them with a high-I^−^ diet, enabling them to produce higher levels of THs than the youngest child and thereby lessening their cognitive deficit. In the studies reported here, we tested this hypothesis by generating a NIS KO mouse model (Fig. 1), which recapitulates the conditions of ITD, and which provides evidence that even in the absence of NIS expression (Fig. 2), extremely high dietary I^−^ can partially restore TH biosynthesis (Fig. 1e). Consistent with these findings, studies of ITD patients that preceded our cloning of NIS^5^ revealed that some of these patients’ symptoms were ameliorated when they were given 14 (or even 100) mg potassium iodide daily^23, 24^, which is ~90 (or ~640) times the daily amount recommended for adults by the Food and Nutrition Board^45^ and the World Health Organization^46^. We recapitulated this condition by feeding NIS KO mice a CD supplying 40 times the recommended minimum amount of I^−^ for rodents^34^.

Here, we formally demonstrated that NIS is the only protein that *actively* accumulates I^−^ in the thyroid, stomach, and salivary glands (Fig. 3a). We also showed that I^−^ can enter the thyroid through non-specific mechanisms, most likely basolaterally expressed Cl^−^ channels or cotransporters. NIS, a specific active transporter that mediates I^−^ accumulation in the thyroid, must have been crucial in evolution, as serum [Cl^−^]s are >10^6^ times higher than serum [I^−^]s (~100 mM vs. ~300 nM)^1^. A higher dietary I^−^ in turn increases serum [I^−^]s, which generates a concentration gradient that allows I^−^ to diffuse into the thyroid follicular cells. A critical mechanism for maintaining this gradient is the upregulation of genes regulated by TSH (Fig. 4d): these genes code for proteins that increase the efficiency of I^−^ organification, which makes the I^−^ gradient favorable for the passive diffusion of I^−^ into the thyroid. TSH has been reported to increase expression only of the protein pendrin, but not of *Slc26a4*, the gene encoding it, *in vitro* ^47^. However, we observed increased levels of *Slc26a4* gene expression in our NIS KO mice (Fig. 4e), which, as shown in Fig. 4c, have higher TSH levels than WT mice.

The overexpression in NIS KO mice of TSH-regulated genes involved in TH biosynthesis is consistent with the notion that, in the absence of NIS, high dietary I^−^ partially rescues TH biosynthesis. This is likely because an inwardly directed I^−^ concentration gradient is generated, allowing I^−^ to diffuse passively into the thyroid follicular cells through non-specific routes, followed by the immediate oxidation of I^−^ to iodine and the incorporation of this iodine into TG by TPO. As previously proposed^36^, when there is only a small amount of iodine available, the marked downregulation of TG may be a mechanism for preventing it from being incorporated into tyrosine residues that will not end up being part of T_4_ (or T_3_) molecules.

Mitochondria are the main source of ROS in the cell^48^. The mitochondrial electron transport chain generates superoxide radicals (O_2_^−^). SODs promptly convert O_2_^−^ into H_2_O_2_ and O_2_^48, 49^. Although ROS are key second messengers in several signal transduction pathways^50, 51^, an excess of them can lead to oxidative stress characterized by increased cellular macromolecule damage, as often occurs in aging^52^. THs regulate mitochondrial activity, and severe hyperthyroidism is associated with cellular damage due to increased ROS production^49^. Reduced thyroid function, by contrast, is associated with longevity in both human and animal models^53–55^. It has been hypothesized that THs stimulate mitochondrial activity, which contributes to increased ROS production and spurs on the aging process^53, 55^. Here, we measured FR levels in BAT, liver, and thyroid under hypothyroid conditions. BAT is rich in mitochondria, important for thermoregulation, and one of the main targets of THs. Under hypothyroid conditions, we found that BAT displays significantly reduced FR and H_2_O_2_ levels (Fig. 5d,e). However, the FR–related genes were not downregulated, and *Sod1*, *Sod2*, and *Cat* were even upregulated (Fig. 5f). THs also regulate the expression of metabolic genes in the liver, which plays a central role in glucose and lipid metabolism^31, 56, 57^. In the liver, we found that FR and H_2_O_2_ levels were reduced (Fig. 5g,h), although not significantly, and several FR–related genes were upregulated (Fig. 5i). Our results indicate that hypothyroidism affects genes involved in FR metabolism.

The H_2_O_2_-rich environment in the thyroid is key for TH biosynthesis, as H_2_O_2_ is essential for I^−^ oxidation. TSH and I^−^ have antagonistic effects on H_2_O_2_ production in thyrocytes. TSH induces H_2_O_2_ production, whereas I^−^ inhibits it^58, 59^. These opposing effects guarantee protection against oxidative stress in euthyroid conditions when TSH levels are within the physiological range and I^−^ levels are adequate, but allow improved I^−^ organification in hypothyroid conditions when TSH is increased and I^−^ is scarce. It is not surprising that hypothyroid conditions increase metabolism in the thyroid. Thyrocytes are forced to proliferate by chronic TSH stimulation, an adaptive response that attempts to compensate for reduced TH levels. This may be the cause of the increased FR and H_2_O_2_ levels we observed (Fig. 5a,b). DUOX 1 and 2 are part of the TH biosynthetic pathway: they produce H_2_O_2_, which is crucial for I^−^ organification. Interestingly, *Duox* genes are strongly downregulated in the thyroids of hypothyroid mice (Fig. 4d). This may be a mechanism to protect the thyroid from the higher H_2_O_2_ levels caused by the increased metabolic activity of the thyrocytes. Although the oxidative environment produced by hypothyroidism may be interpreted as an adaptive response aimed at making I^−^ oxidation more efficient, it may be dangerous, as it may damage macromolecules, including lipids, proteins and DNA^40, 60^. This oxidative environment can, in the long term, cause DNA mutations that can lead to thyroid cancer^61^.

In summary, we report here, for the first time, the generation of a constitutive NIS KO mouse model, which is characterized by hypothyroidism, with reduced TH and increased TSH levels. We have demonstrated that I^−^ can enter the thyroid via mechanisms other than NIS, and that, in hypothyroidism, peripheral tissues display a less oxidative intracellular environment, whereas the thyroid, stimulated by TSH, evinces an increased intracellular concentration of free radicals.

## Materials and Methods

### Animals

Mouse protocols were approved by the Yale University International Animal Care and Use Committee (IACUC). All experiments were performed in accordance with relevant guidelines and regulations. Five-to-seven-month-old male C57BL/6 J/N-A mice with mixed genetic backgrounds were used unless otherwise specified. Sex-, age-, and diet-matched WT mice with the same genetic background were used as controls. Mice were fed a chow diet (CD) (Harlan 2018, which provides 6 µg of I^−^ per gram) up until they were switched to the minimal iodide diet (MID) (Harlan TD.150677, which provides 0.15 µg of I^−^ per gram), two weeks prior to the metabolic studies. Both the CD and the MID provide ~20% of calories from protein, ~60% from carbohydrates, and ~20% from fat. NIS KO mice were generated by following the standard procedure reported before^62^.

*Slc5a5*-targeted embryonic stem (ES) cells harboring the C57BL/6N-A^tm1Brd^ (http://www.informatics.jax.org/allele/key/814110) genetic background were injected into C57BL/6 J blastocysts to obtain chimeric mice. Chimeras were crossed with B6.Cg-Tg(ACTFLPe)9205Dym/J mice (https://www.jax.org/strain/005703) harboring the C57BL/6 J genetic background. Litters positive for the *Slc5a5* targeted allele were crossed with FVB/N-Tg(ACTB-cre)2Mrt/J mice (https://www.jax.org/strain/003376), which had been backcrossed for over 20 generations with C57BL/6 J mice. NIS KO mice were crossed with C57BL/6 J WT mice to expand the colony.

### Western blot

SDS-PAGE, electrotransfer to PVDF membranes, and immunoblotting were carried out as described^6^. Membranes were incubated with 4 nM of an affinity-purified polyclonal anti-rat NIS Ab (diluted 1:2000 in TBST 5% Non-Fat Omniblok milk Americanbio AB10109) directed against 16 residues of the cytosolic NIS carboxyl terminus^6^. Equal loading was assessed by stripping and reprobing the same blot with 0.5 µg/ml monoclonal anti-β-actin Ab (Cell Signaling 4970) diluted 1:1000 in TBST 5% Non-Fat Omniblok milk. Horseradish peroxidase (HRP)-linked secondary anti-mouse and anti-rabbit Abs were from Jackson ImmunoResearch (West Grove, PA, USA). Proteins were visualized using the enhanced chemiluminescence western blot detection system (Amersham Biosciences).

### Immunohistochemistry

Organs were fixed overnight in 10% neutral buffered formalin prior to paraffin inclusion^63^.

5-μm sections were deparaffinated in xylene and rehydrated through graded alcohols. Then antigen retrieval was performed at 95 °C for 15 min with 10 mM citrate buffer solution. After cooling slides, endogenous peroxidase was quenched with 3% hydrogen peroxide. Slides were incubated with 5% goat serum. Slides were incubated overnight with an affinity purified anti-rat NIS Ab^6^ diluted 1:3000 in PBS 0.5% BSA. The remainder of the immunoperoxidase procedure was carried out according to the supplier’s instructions provided in the EnVision System-HRP (DAKO K4010). Slides were analyzed by light microscopy.

### MicroSPECT/CT

At each imaging session animals were injected with ^99m^TcO_4_^−^ [0.962 ± 0.137 mCi (mean ± SEM)] via the tail vein for microSPECT/CT imaging using a dedicated high-resolution small-animal hybrid imaging system (GammaMedica, X-SPECT)^33^. One and a half hours following injection of ^99m^TcO_4_^−^, ungated CT imaging was performed (50 kVp/800 μA; projections, 512) to identify the anatomic structures. Immediately following the CT scan, microSPECT images were acquired using the following acquisition parameters: 32 projections, 60 seconds per projection, and 140 keV photopeak ± 20% window. Imaging occurred under light isoflurane anesthesia (1.5–2.0% isoflurane/98.0–98.5% oxygen).

### MicroSPECT/CT Image Reconstruction and Analyses

Reconstructed microSPECT images were reoriented according to the CT anatomic images and external point sources, fused, and exported in “Analyze” format (Analyze, Mayo Clinic) for further processing using Amide Medical Imaging Data Examiner (amide.sf.net)^33^. Images were corrected for injected dose and decay from injection time and displayed as % injected dose/cc.

### ^99m^TcO_4_^−^ quantitation in tissues

Mice were euthanized with saturated KCl (1–2 mmol/kg) and their tissues rapidly extracted. Each tissue piece’s radioactivity was measured by gamma well counting (Cobra Packard). Raw counts were corrected for spill-up/spill-down, background, decay, and weight. Corrected counts were converted to mCi/g with the use of previously determined counter efficiency. Activity in each tissue segment was then calculated as percentage of injected dose (%ID) by correcting for decay to the time of radiotracer injection. The calculated %ID was computed by dividing corrected tissue counts (mCi/g) by the corrected injected dose (mCi) and expressing it as %ID per gram tissue (%ID/g)^33^.

### Hormone, free radical, and H_2_O_2_ measurements

Hormone levels were determined using age-matched male mice. TH levels were quantitated with the Thyroxine (T_4_) ELISA (Mouse/Rat) Kit (Sigma SE120090 and Calbiotech T4044T)^64, 65^ and the Triiodothyronine (T_3_) ELISA (Mouse/Rat) Kit (Sigma SE120091 and Calbiotech T3043T)^65, 66^.

TSH levels were quantitated using the Milliplex map mouse pituitary magnetic bead panel (Millipore MPTMAG-49K-01), following the manufacturer’s instructions.

Free radicals and H_2_O_2_ were measured using the OxiSelect^TM^ *in Vitro* ROS/RNS Assay kit (Green fluorescence; Cell Biolabs Inc STA-347)^67–69^. The proprietary non-fluorescent dichlorodihydrofluorescein DiOxyQ (DCFH-DiOxyQ) probe was primed to form the non-fluorescent DCFH-DiOxy and then converted to the non-fluorescent DCFH. DCFH can react with ROS and H_2_O_2_ to form fluorescent DCF. Tissues were homogenized in PBS and incubated with DCFH and catalytic agents. The fluorescence intensity was analyzed using a Tecan infinite M1000 with the excitation at 480 nm and emission at 530 nm. Data were normalized against a DCF or an H_2_O_2_ standard curve. All kits were used according to the manufacturer’s instructions.

### Real Time PCR

RNA from frozen dissected tissues was extracted by using Trizol reagent (Ambion 15596026). cDNA was synthesized by using the iScript cDNA Synthesis Kit (Biorad 1708891). Five ng of cDNA were amplified by using Power SYBR® Green PCR Master Mix (Applied Biosystems 4368577), according to the manufacturer’s instructions. The Light Cycler 480 System (Roche Life Science) was used to carry out amplification. For each sample, the expression of the genes of interest was normalized to the expression of *Rn18S* measured under the same conditions. Specific primers for the analyzed genes were designed by using Primer-BLAST http://www.ncbi.nlm.nih.gov/tools/primer-blast/ if not otherwise specified. Primer sequences are given in Supplementary Table 1.

### Statistical analysis

All the quantitative measurements represent the average ± SEM of 4–8 mice per group if not differently indicated. The differences between the groups were determined using a two-tailed Student’s T-test. All calculations were carried out in Microsoft Excel. *Indicates *P* < 0.05, ^#^ *P* < 0.01.

## Electronic supplementary material

Supplementary information
