# Supplementary material for: An extremely high dietary iodide supply forestalls severe hypothyroidism in Na+/I− symporter (NIS) knockout mice
Source: Sci Rep. 2017 Jul 13;7:5329. doi: 10.1038/s41598-017-04326-z (PMC5509730; doi:10.1038/s41598-017-04326-z)
Supplement: Supplementary file 1 — Supplementary information [file 41598_2017_4326_MOESM1_ESM.pdf]

An extremely high dietary iodide supply forestalls severe hypothyroidism in Na^{+}/I^{-} symporter (NIS) knockout mice

Giuseppe Ferrandino, Rachel R. Kaspari, Andrea Reyna-Neyra, Nabil E. Boutagy, Albert J. Sinusas, and Nancy Carrasco

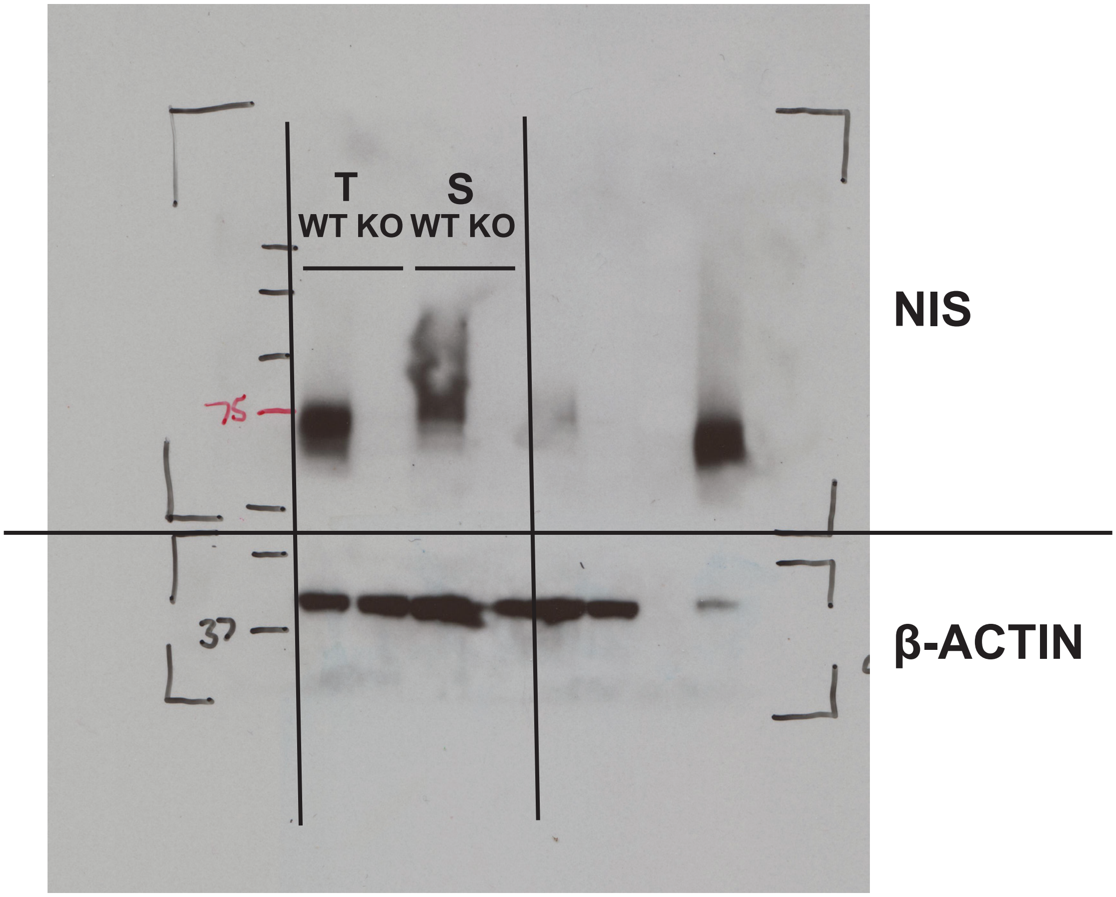

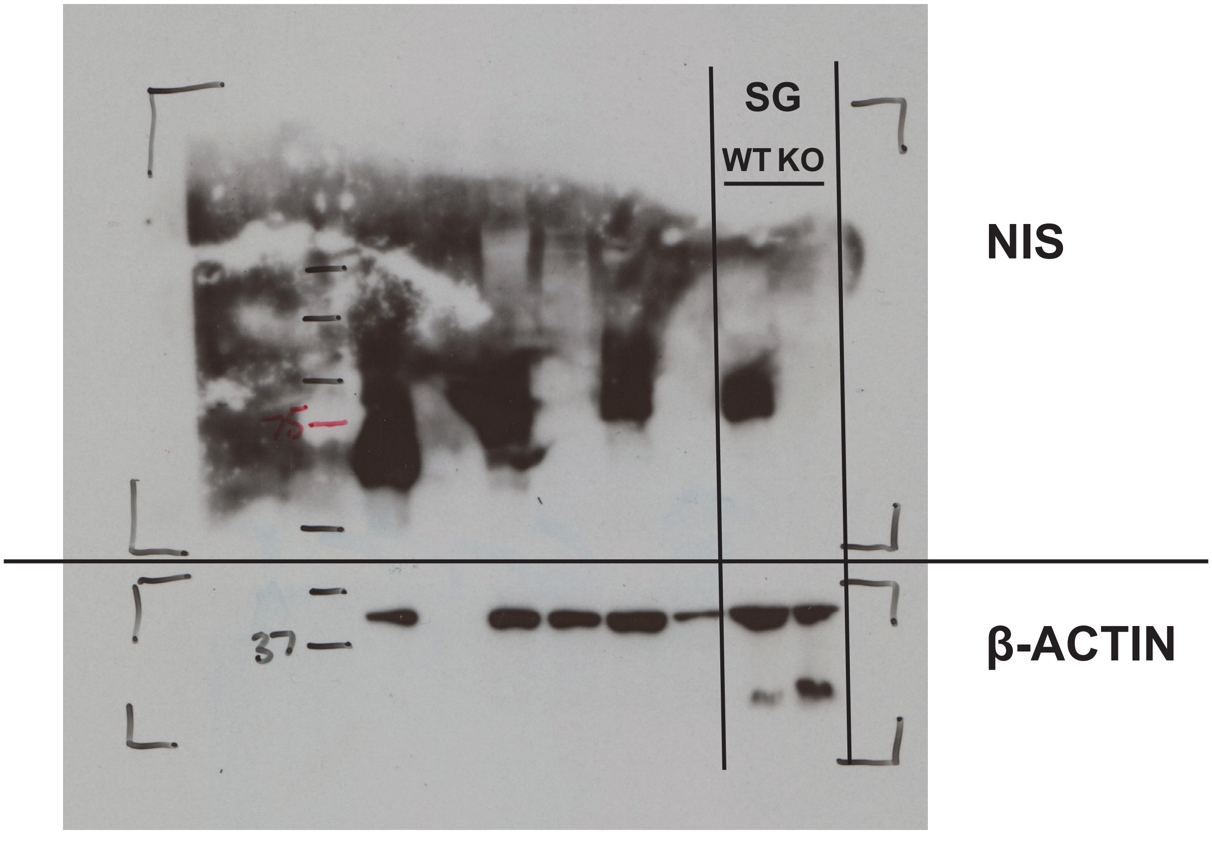

Supplementary Figure 1. Full images of the Western blots of NIS expression in WT and NIS KO mice fed a CD.

**Table 1. List of primers used for quantitative RT-PCR**			
Primer name	Gene ID / primer bank ID	Primer sequence	Primer efficiency
SLC5a5 fw	114479	CCAGTTGCTCAATTCGCTGC	2
SLC5a5 rev		CGCAGTTCTAGGTACTGGTAGG	
TSHr fw	22095	GCGCTTTTAAGAACCAGAAGAA	2
TSHr rev		GGTTCCGGATACTGCTCTCA	
TPO fw	22018	TTTGGAGCCAGACATTGGCA	1.8
TPO rev		GCAGCCTTGGGCTGATGATA	
Dehal fw	70337	ACCCGATACCCAGAACAGGA	2
Dehal rev		CGTGCTCGCTGCTGATAAAC	
Tg fw	21819	CCCAGACTACTTCAGTCCGC	2
Tg rev		ACAGCTATCGAACGCAGGAG	
Duox1 fw	99439 / 26331898a1	AAAACACCAGGAACGGATTGT[1]	2
Duox 1 rev		AGAAGACATTGGGCTGTAGGG[1]	
Duox2 fw	214593 / 26348219a1	AAGTTCAAGCAGTACAAGCGAT[1]	2
Duox 2 rev		TAGGCACGGTCTGCAAACAG[1]	
Clc5 fw	12728	GTGCCTGGTTACACACAACG	1.9
Clc5 rev		CTGTGCTATATGCTTTAACACATCC	
Cftr fw	12638	CAGCAGCTCAAACAACTGGA	2
Cftr rev		TGTCACAAGGTGGGTGAAAA	
Slc26a4 fw	23985	GCTGGCCTCATCTCAGCTG[2]	2
Slc26a4 rev		GCAAGGGTTCCAGAAGCCT[2]	
Slc5a8 fw	216225	CTTATGGGCGGTCGCAGTAT	2
Slc5a8 rev		AAAACGGTAGACCTCGGCAG	
Nfe2l2 fw	18024	AGGTTGCCCACATTCCCAAA	1.8
Nfe2l2 rev		CCTGATGAGGGGCAGTGAAG	
Gpx1 fw	14775	TGCAATCAGTTCGGACACCA	1.8
Gpx1 rev		AAGGTAAAGAGCGGGTGAGC	
Sod 1 fw	20655	GGAACCATCCACTTCGAGCA	1.8
Sod 1 rev		CCCATGCTGGCCTTCAGTTA	
Sod 2 fw	20656	GTAGGGCCTGTCCGATGATG	2
Sod 2 rev		CGCTACTGAGAAAGGTGCCA	
Catalase fw	12359	AGCGACCAGATGAAGCAGTG	1.8
Catalase rev		TCCGCTCTCTGTCAAAGTGTG	
Txn1 fw	22166	GCGCTCCGCCCTATTTCTAT	2
Txn1 rev		TCACCATTTTGGCTGTTGCG	
Gstp1 fw	14870	GCGGCAAATATGTCACCCTCA	1.9
Gstp1 rev		GAAAGCTTTGCCTCCCTGGT	
Gsta2 fw	14858	GAGCTTGATGCCAGCCTTCT	1.8
Gsta2 rev		GCATCCAAGGGAGGCTTTCT	
Dio1 fw	13370	CCCTGGTGTTGAACTTTGGC	1.8
Dio1 rev		TGAGGAAATCGGCTGTGGA	
18s fw	19791	AACCCGTTGAACCCCATT	2
18 s rev		CCATCCAATCGGTAGTAGCG	
Slc16a2 fw	20502 / 27370260a1	CGGCTGGATAGTGGTGTTTG	2
Slc16a2 rev		CAGAGTTATGGATGCCGAAGATG	

Supplementary references

1. Spandidos, A., Wang, X.W., Wang, H.J. & Seed, B. PrimerBank: a resource of human and mouse PCR primer pairs for gene expression detection and quantification. *Nucleic Acids Res* **38**, D792-D799 (2010).
2. Wall, S.M.*, et al.* Localization of pendrin in mouse kidney. *Am J Physiol-Renal* **284**, F229-F241 (2003).
